# Growth hormone-releasing hormone antagonist inhibits the invasiveness of human endometrial cancer cells by down-regulating twist and N-cadherin expression

**DOI:** 10.18632/oncotarget.13877

**Published:** 2016-12-10

**Authors:** Hsien-Ming Wu, Hong-Yuan Huang, Andrew V Schally, Angel Chao, Hung-Hsueh Chou, Peter C.K. Leung, Hsin-Shih Wang

**Affiliations:** ^1^ Department of Obstetrics and Gynecology, Chang Gung Memorial Hospital Linkou Medical Center, Chang Gung University School of Medicine, Taoyuan, Taiwan R.O.C. 333; ^2^ Department of Obstetrics and Gynecology, University of British Columbia, Vancouver, British Columbia, Canada V6H3V5; ^3^ Veterans Affairs Medical Center and Departments of Pathology and Medicine, Division of Hematology/Oncology, University of Miami Miller School of Medicine, Miami, FL 33125, USA

**Keywords:** GHRH antagonist, endometrial cancer, invasion, twist, N-cadherin

## Abstract

More than 25% of patients diagnosed with endometrial carcinoma have invasive primary cancer accompanied by metastases. Growth hormone-releasing hormone (GHRH) plays an important role in reproduction. Here, we examined the effect of a GHRH antagonist on the motility of endometrial cancer cells and the mechanisms of action of the antagonist in endometrial cancer. Western blotting and immunohistochemistry (IHC) were used to determine the expression of the GHRH receptor protein. The activity of Twist and N-cadherin was determined by Western blotting. Cell motility was assessed by an invasion and migration assay. GHRH receptor siRNA was applied to knockdown the GHRH receptor in endometrial cancer cells. The GHRH antagonist inhibited cell motility in a dose-dependent manner. The GHRH antagonist inhibited cell motility and suppressed the expression of Twist and N-cadherin, and the suppression was abolished by GHRH receptor siRNA pretreatment. Moreover, the inhibition of Twist and N-cadherin with Twist siRNA and N-cadherin siRNA, respectively, suppressed cell motility. Our study indicates that the GHRH antagonist inhibited the cell motility of endometrial cancer cells through the GHRH receptor via the suppression of Twist and N-cadherin. Our findings represent a new concept in the mechanism of GHRH antagonist-suppressed cell motility in endometrial cancer cells and suggest the possibility of exploring GHRH antagonists as potential therapeutics for the treatment of human endometrial cancer.

## INTRODUCTION

Endometrial cancer is the most common, and second most lethal, gynecological malignancy. Despite aggressive treatment, the 5-year survival rate is still less than 20% [1]. In endometrial cancer, most of deaths associated are caused by chemotherapy-resistant metastases. Therefore, investigation into the molecular mechanisms behind endometrial cancer metastasis would provide insight for the development of improved therapies. [2]. To date, the underlying molecular mechanisms that involved in the pathogenesis of endometrial cancer are still not well understood. In mammals, growth hormone-releasing hormone (GHRH) is secreted by the hypothalamus. In pituitary, GHRH binds to its receptor, GHRH receptor (GHRH-R), to stimulate the synthesis and secretion of growth hormone [3, 4]. It has been shown that GHRH can be secreted by many extrahypothalamic tissues and tumor cells. In human cancers, GHRH has been reported to function as an antocrine regulatory factor [3–6]. Our previous study has demonstrated that GHRH and GHRH-R are expressed in human endometrial cancer cells [6]. However, to the best of our knowledge, the effect of GHRH on the invasion of human endometrial cancer cells remains unknown, and the underlying mechanisms by which GHRH regulates the endometrial cancer cell invasion need to be delineated. N-cadherin is a member of the superfamily of integral membrane glycoproteins that regulates cell adhesion and cell motility [7, 8]. N-cadherin plays an important role in promoting invasion during cancer progression [9, 10]. Twist, a helix-loop-helix transcription factor, is known to play a key role in regulation of cell invasion and tumor metastasis [11, 12]. Importantly, Twist has been characterized as a critical transcription factor that regulates the expression of N-cadherin in cancer cells [13, 14]. In different types of cancer, treatment with GHRH antagonist decreases cell migration and invasion [15, 16]. Based on these studies, we hypothesized that GHRH antagonist inhibits human endometrial cancer cell migration and invasion by down-regulating the expression of N-cadherin. In the present study, we examined the effect of GHRH antagonist on the cell migration and invasion of two human endometrial cancer cell lines, Ishikawa and ECC-1, as well as the underlying molecular mechanisms of action involved. Our data suggest that GHRH antagonist might be a potential molecule for the clinical treatment of human endometrial cancer.

## RESULTS

### GHRH receptor (GHRH-R) and GHRH are expressed in human endometrial cancer cells

**Figure 1:**
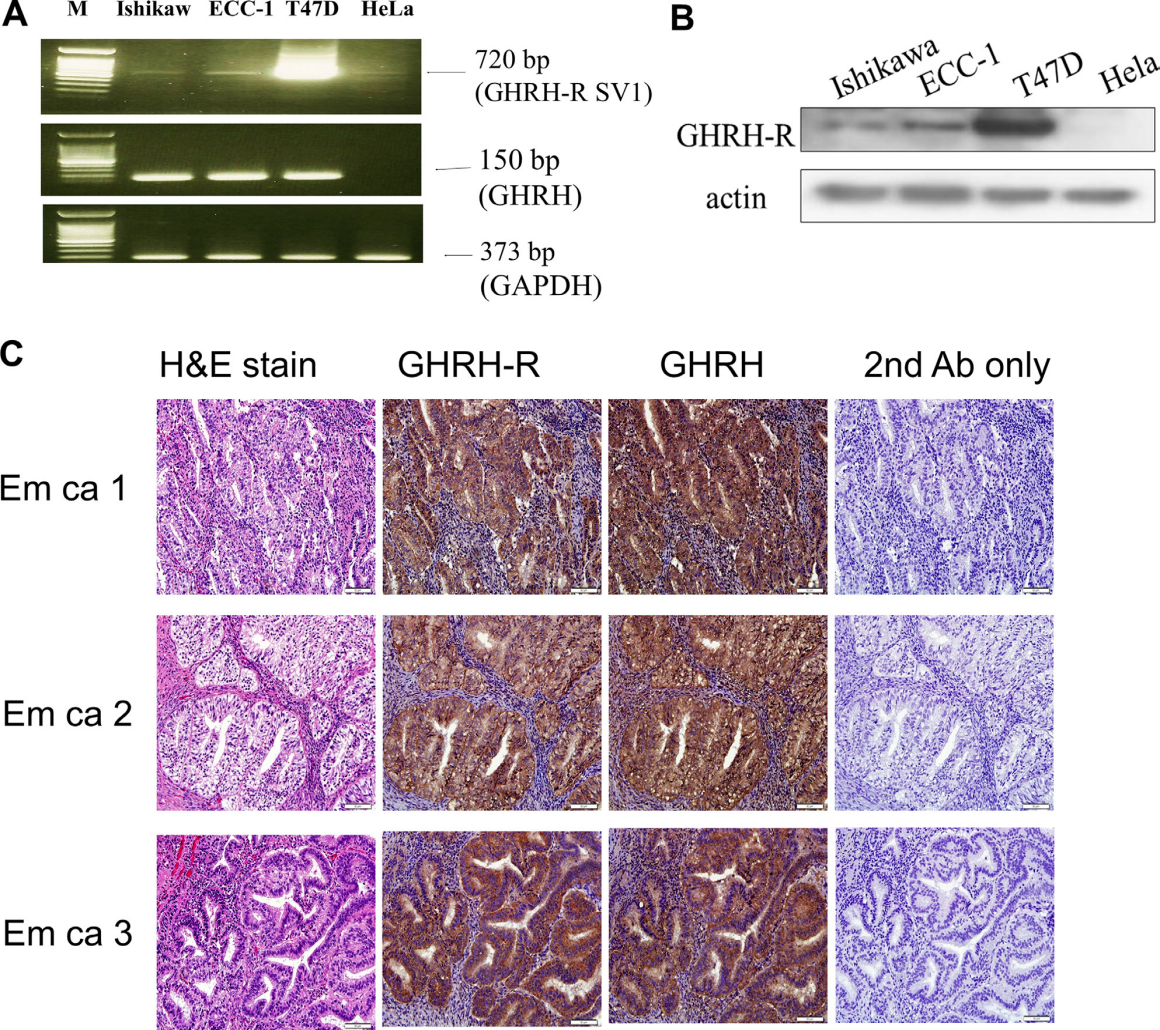
Expression of GHRH receptor (GHRH-R) and GHRH in human endometrial cancer (**A**) RT-PCR analysis of GHRH-R SV1 and GHRH mRNA expression in Ishikawa and ECC-1 endometrial cancer cells. T47D breast cancer cells served as a positive control, and HeLa cells served as a negative control. Amplification of GAPDH was performed to ensure equal loading. The size of the PCR products, predicted on the basis of their cDNA sequence, was 720 bp for GHRH-R SV1, 150 bp for GHRH, and 373 bp for GAPDH. The results are representative of three independent experiments. (**B**) GHRH-R levels in Ishikawa and ECC-1 cells were examined by Western blotting assays. T47D cells served as a positive control and HeLa cells served as a negative control. (**C**) GHRH-R was stained brown in the second of the four columns depicting human endometrial cancer tissue sections. GHRH was stained brown in the third of the four columns depicting human endometrial cancer tissue sections. Sections were counterstained with hematoxylin to show the nuclei in column 1 of the four columns depicting human endometrial cancer tissue sections. Sections were stained without the GHRH-R and GHRH antibodies as a negative control in the fourth of the four columns depicting human endometrial cancer tissue sections. Micrographs were taken with a 40× objective lens. Scale bars represent 20 μm.

To examine the expression of GHRH-R in endometrial cancer, two human endometrial cancer cell lines, Ishikawa and ECC-1, were used. As shown in Figure 1A, GHRH-R splice variant 1 (SV1) and GHRH mRNA expressions were detected in both Ishikawa and ECC-1 cells. T47D breast cancer cells and HeLa cells were used as a positive and negative control, respectively. Western blotting results confirmed the expression of GHRH-R protein in both Ishikawa and ECC-1 cells (Figure 1B). Moreover, immunohistochemical analyses showed that GHRH-R and GHRH are expressed in the tumor tissues of human endometrial cancer (Figure 1C).

### GHRH antagonist inhibits human endometrial cancer cell migration and invasion

**Figure 2:**
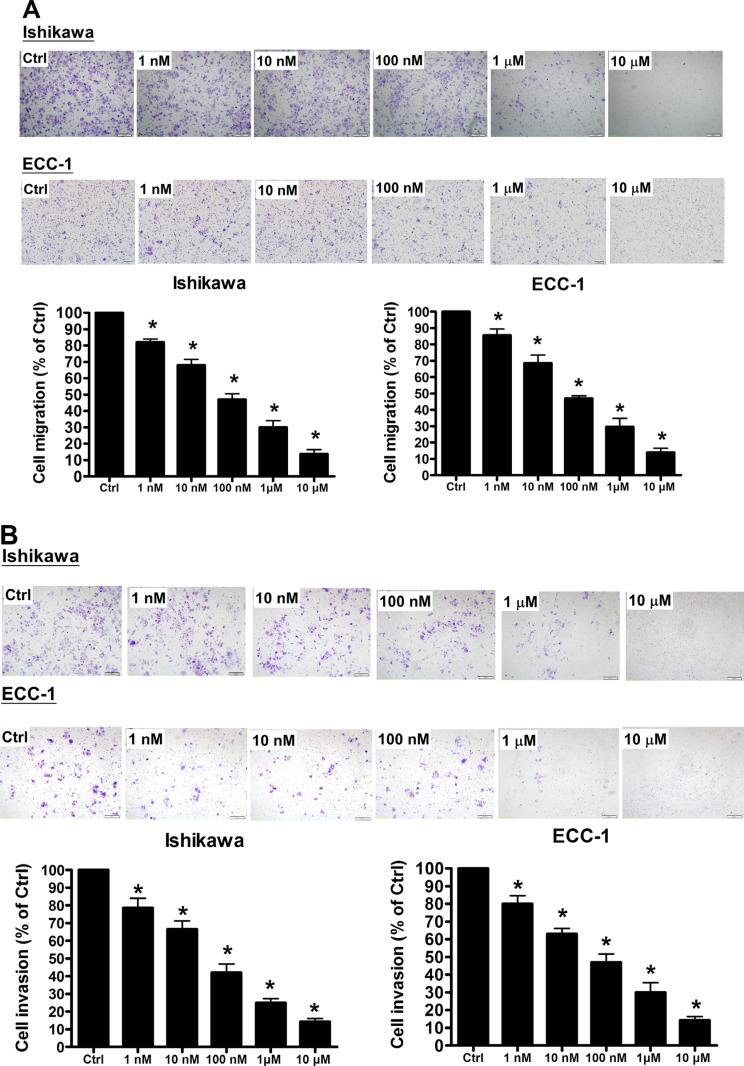
GHRH antagonist inhibits endometrial cancer cell migration and invasion (**A**) Using the Transwell migration assay, the migration and invasion capability of endometrial cancer cells was assessed. The GHRH antagonist suppressed the migration of endometrial cancer cells through an uncoated porous filter in a dose-dependent manner at concentrations of 1 nM to 10 μM, with a maximal effect at 10 μM. (**B**) Endometrial cancer cells were seeded onto a Matrigel-pre-coated filter in Transwell chambers in the presence or absence of increasing concentrations of GHRH antagonist (1 nM to 10 μM, as indicated). After 24 (migration) and 48 (invasion) h of incubation, cells on the upper side of the filter were removed, and the cells that migrated or invaded were fixed, stained, and counted. On the left, representative pictures are shown. In the columns, the mean number of migrated or invaded cells in five fields of triplicate wells from three independent experiments is presented. Bars, SD; *p < 0.05, versus control.

To examine whether GHRH-R is involved in regulation of human endometrial cancer cell migration and invasion, Ishikawa and ECC-1 were treated with a GHRH antagonist, MIA-602. As shown in Figure 2A, treatment with MIA-602 (1 nM-10 μM) significantly inhibited the cell migration in a dose-dependent manner in both Ishikawa and ECC-1 cells. In addition, invasion assay results showed that treatment with MIA-602 also inhibited the cell invasion of Ishikawa and ECC-1 cells in a dose-dependent manner (Figure 2B). Taken together, these results clearly indicated that GHRH antagonist exhibited an inhibitory effect on the cell migration and invasion in human endometrial cancer.

### Knockdown of Twist decreases human endometrial cancer cell migration and invasion

**Figure 3:**
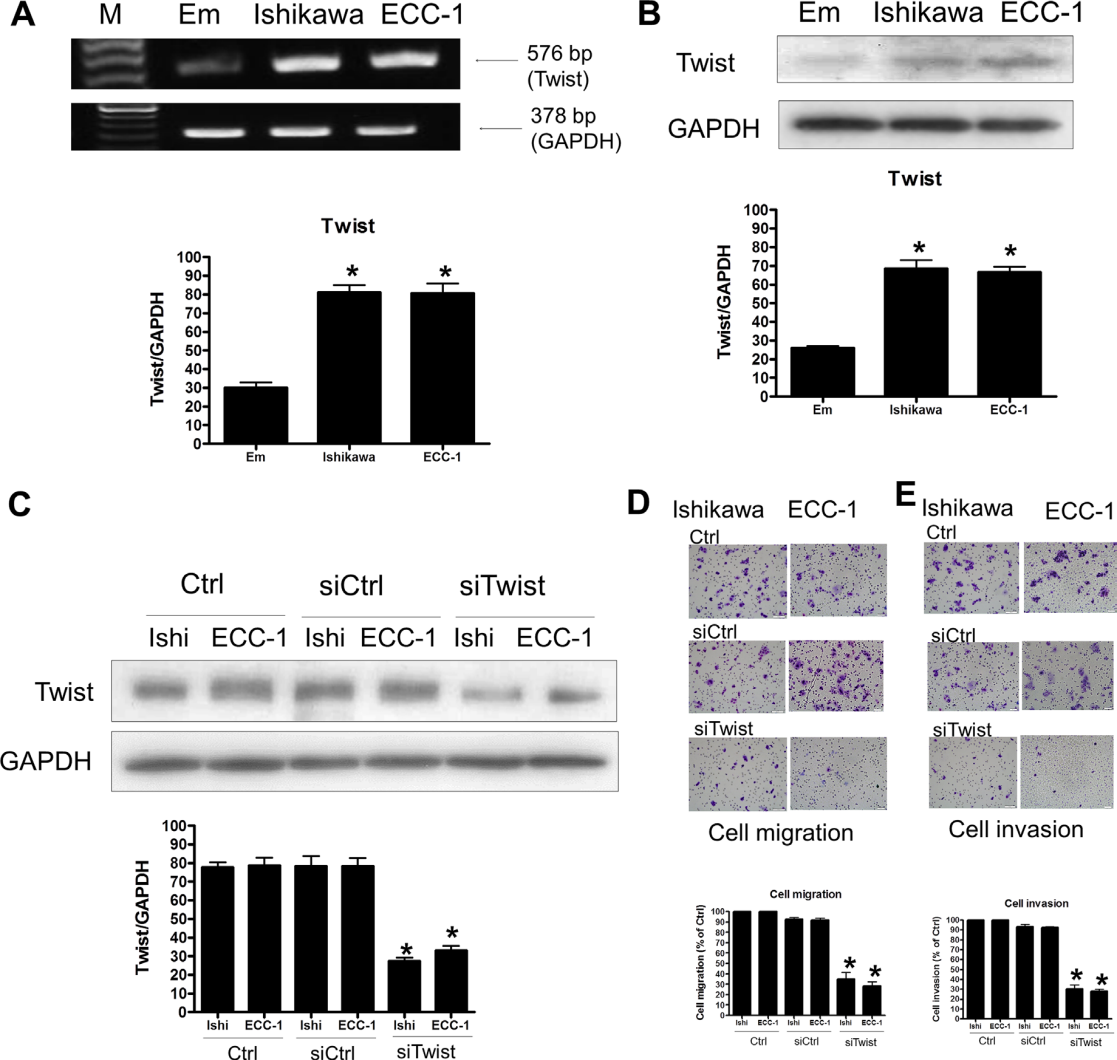
The effects of Twist signaling in endometrial cancer cells (**A**) Semiquantitative RT-PCR analysis of Twist mRNA levels in endometrium (Em), Ishikawa, and ECC-1 endometrial cancer cells. A 100-bp ladder is shown in lane M (marker) with the size of the target cDNA indicated at the right. Absorbance values for Twist mRNA were standardized to GAPDH mRNA levels. The results are expressed as the mean ± SEM of three independent experiments. (*p < 0.05, versus endometrium). (**B**) Western blotting analysis of Twist protein expression in normal endometrium, Ishikawa and ECC-1 endometrial cancer cells. Absorbance values of the Twist protein were standardized to GAPDH protein levels. The results are expressed as the mean ± SEM of three independent experiments. (*p < 0.05, versus endometrium). (**C**) Effects of human Twist siRNA (siTwist) transfection on endometrial cancer cells. Twist levels were monitored by Western blotting. The endometrial cancer cells were transfected with human siTwist or scrambled siRNA (siCtrl) for one day with Lipofectamine RNAiMAX. (**D**) The effects of siTwist transfection on endometrial cancer cell migration. Cells were transfected with siTwist and siCtrl for 24 h. The cell motility was assessed with the migration assay. The results are expressed as the mean ± SEM of three independent experiments. (*p < 0.05, versus control). (**E**) The effects of siTwist transfection on endometrial cancer cell invasion. Cells were transfected with siTwist and siCtrl for 48 h. The cell motility was assessed with the invasion assay. The results are expressed as the mean ± SEM of three independent experiments. (*p < 0.05, versus control).

To investigate the role of Twist in human endometrial cancer migration and invasion, we first examined the expression of Twist in Ishikawa and ECC-1 cells. As shown in Figure 3A, Twist mRNA expression was detected in both Ishikawa and ECC-1 cells. Interestingly, compared to the normal endometrium, Twist mRNA levels were up-regulated in Ishikawa and ECC-1 cells. Western blotting results further confirmed the up-regulation of Twist protein levels in Ishikawa and ECC-1 cells compared to the normal endometrium (Figure 3B). Transfection cells with Twist siRNA knocked down the endogenous expression levels of Twist (Figure 3C). In addition, siRNA-mediated knockdown of Twist decreased the basal cell migration of Ishikawa and ECC-1 cells (Figure 3D). Moreover, the basal levels of Ishikawa and ECC-1 cell invasion were decreased by Twist knockdown (Figure 3E).

### N-cadherin knockdown decreases human endometrial cancer cells migration and invasion

**Figure 4:**
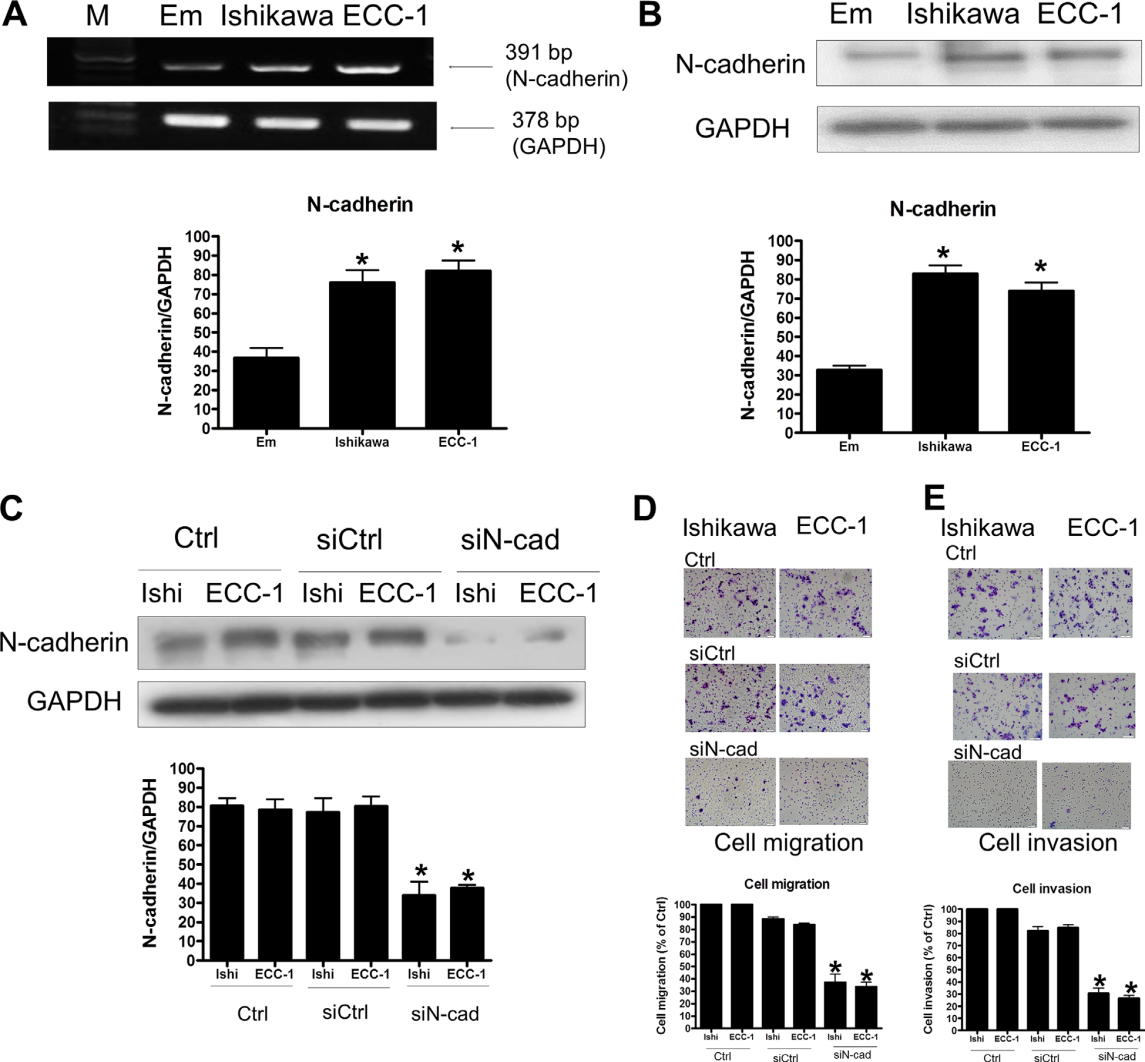
The effects of N-cadherin signaling in endometrial cancer cells (**A**) Semiquantitative RT-PCR analysis of N-cadherin mRNA levels in endometrium (Em), Ishikawa, and ECC-1 endometrial cancer cells. A 100-bp ladder is shown in lane M (marker) with the size of the target cDNA indicated at the right. Absorbance values for N-cadherin mRNA were standardized to GAPDH mRNA levels. The results are expressed as the mean ± SEM of three independent experiments. (*p < 0.05, versus endometrium). (**B**) Western blotting analysis of N-cadherin protein expression in normal endometrium, Ishikawa and ECC-1 endometrial cancer cells. Absorbance values for N-cadherin protein were standardized to GAPDH protein levels. The results are expressed as the mean ± SEM of three independent experiments. (*p < 0.05, versus endometrium). (**C**) Effects of human N-cadherin siRNA (siN-cad) transfection on endometrial cancer cells. N-cadherin levels were monitored by Western blotting. The endometrial cancer cells were transfected with human siN-cadherin or scrambled siRNA (siCtrl) for one day with Lipofectamine RNAiMAX. (**D**) The effects of siN-cad transfection on endometrial cancer cell migration. The cells were transfected with siN-cad and siCtrl for 24 h. Cell motility was assessed with the migration assay. The results are expressed as the mean ± SEM of three independent experiments. (*p < 0.05, versus control). (**E**) The effects of siN-cad transfection on endometrial cancer cell invasion. The cells were transfected with siN-cad and siCtrl for 48 h. Cell motility was assessed with the invasion assay. The results are expressed as the mean ± SEM of three independent experiments. (*p < 0.05, versus control).

Given the importance of Twist in regulation of N-cadherin expression, we next examined whether expression of N-cadherin affects human endometrial cancer migration and invasion. RT-PCR and Western blotting analyses showed that N-cadherin mRNA and protein levels were detected in both Ishikawa and ECC-1 cells. Similar to the results of Twist, N-cadherin expression levels were up-regulated in Ishikawa and ECC-1 cells when compared to the normal endometrium (Figure 4A and 4B). The siRNA-mediated knockdown approach was used to examine the role of N-cadherin in regulation of endometrial cancer cell migration and invasion. As shown in Figure 4C, N-cadherin siRNA significantly down-regulated endogenous N-cadherin expression. Knockdown of N-cadherin decreased not only basal cell migration but also basal cell invasion of Ishikawa and ECC-1 cells (Figure 4D and 4E). Taken together, these results indicated that N-cadherin expression was required for the human endometrial cancer cell migration and invasion.

### GHRH antagonist inhibits human endometrial cancer cell migration and invasion by down-regulating Twist and N-cadherin expression

**Figure 5:**
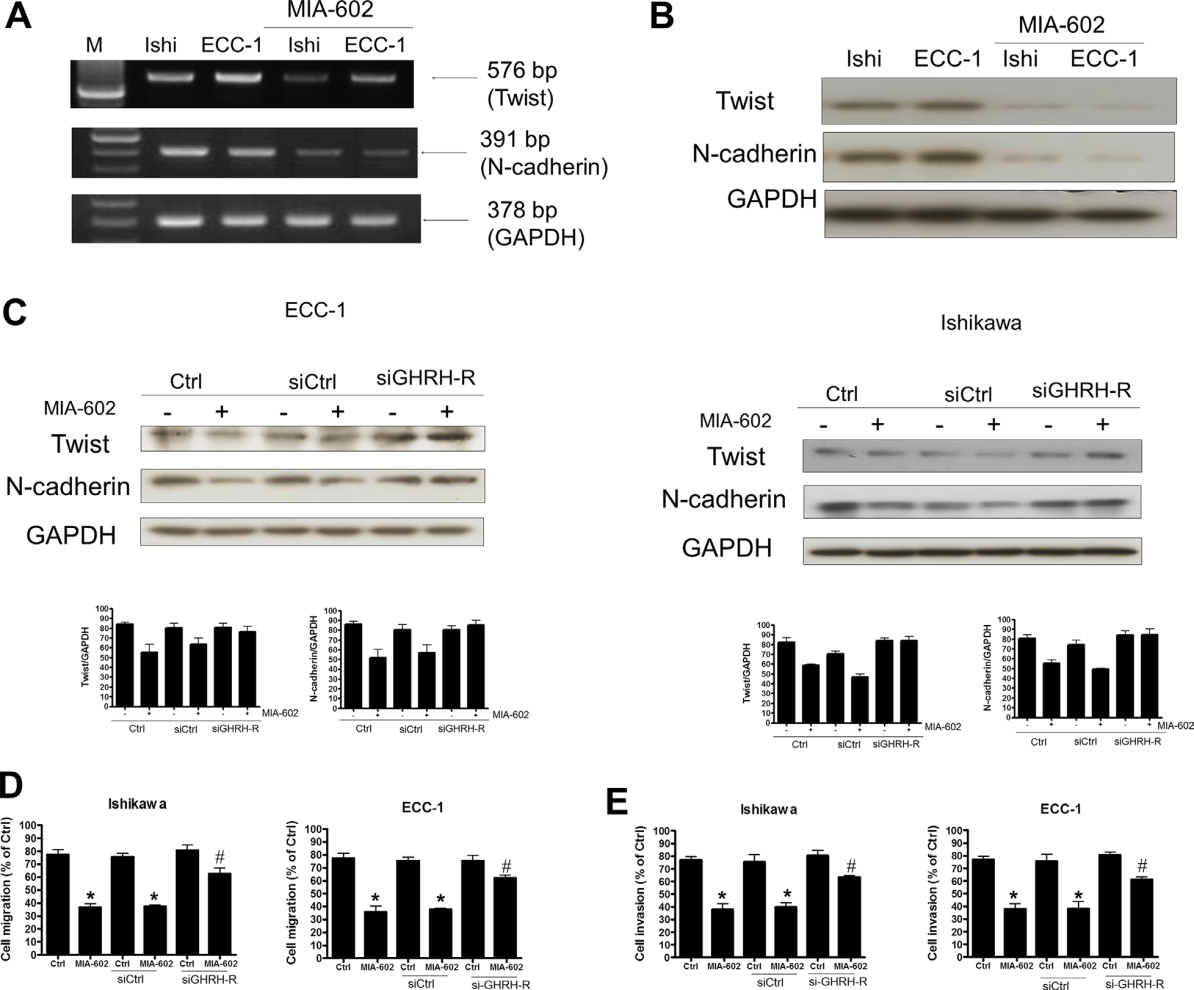
The effects of the GHRH antagonist (MIA-602) on Twist and N-cadherin signaling in endometrial cancer cells (**A**) Effects of MIA-602 on Twist and N-cadherin mRNA expression. Cells were treated with MIA-602 (100 nM), and then semiquantitative RT-PCR analysis of Twist and N-cadherin mRNA levels in Ishikawa and ECC-1 endometrial cancer cells was performed. A 100-bp ladder is shown in lane M (marker) with the size of the target cDNA indicated at the *right*. Absorbance values for Twist and N-cadherin mRNA were standardized to GAPDH mRNA levels. The results are expressed as the mean ± SEM of three independent experiments. (*p < 0.05, versus control). (**B**) Effects of MIA-602 on Twist and N-cadherin protein expression. Western blotting analysis of Twist and N-cadherin protein expression in Ishikawa and ECC-1 endometrial cancer cells. Absorbance values for Twist and N-cadherin protein were standardized to GAPDH protein levels. The results are expressed as the mean ± SEM of three independent experiments. (*p < 0.05, versus control). (**C**) The endometrial cancer cells (right column: ECC-1, left column: Ishikawa) were transfected with either siGHRH-R or siCtrl for 24 h using Lipofectamine RNAiMax, and Twist and N-cadherin activity was monitored by Western blotting, revealing that the MIA-602-induced decreased levels of Twist and N-cadherin activity were rescued following siGHRH-R transfection. GAPDH served as a loading control. (**D**) The effects of GHRH-R siRNA (siGHRH-R) transfection on MIA-602-induced inhibition of cell migration. Ishikawa and ECC-1 cells were transfected with siGHRH-R and treated with MIA-602 (100 nM) every 24 h for 72 h. Cell migration was assessed by the migration assay, and the results indicated that transfection with siGHRH-R rescued endometrial cancer cells from MIA-602-induced inhibition of cell migration. The results are expressed as the mean ± SEM for three independent experiments (*p < 0.05, versus control; #*p* < 0.05, versus MIA-602). (**E**) The effects of GHRH-R siRNA (siGHRH-R) transfection on MIA-602-induced suppression of cell invasion. Ishikawa and ECC-1 cells were transfected with siGHRH-R and treated with MIA-602 (100 nM) every 24 h for 72 h. Cell invasion was assessed by the invasion assay, and the results indicated that transfection with siGHRH-R rescued endometrial cancer cells from MIA-602-induced suppression of cell invasion. The results are expressed as the mean ± SEM for three independent experiments (*p < 0.05, versus control; #*p* < 0.05, versus MIA-602).

**Figure 6:**
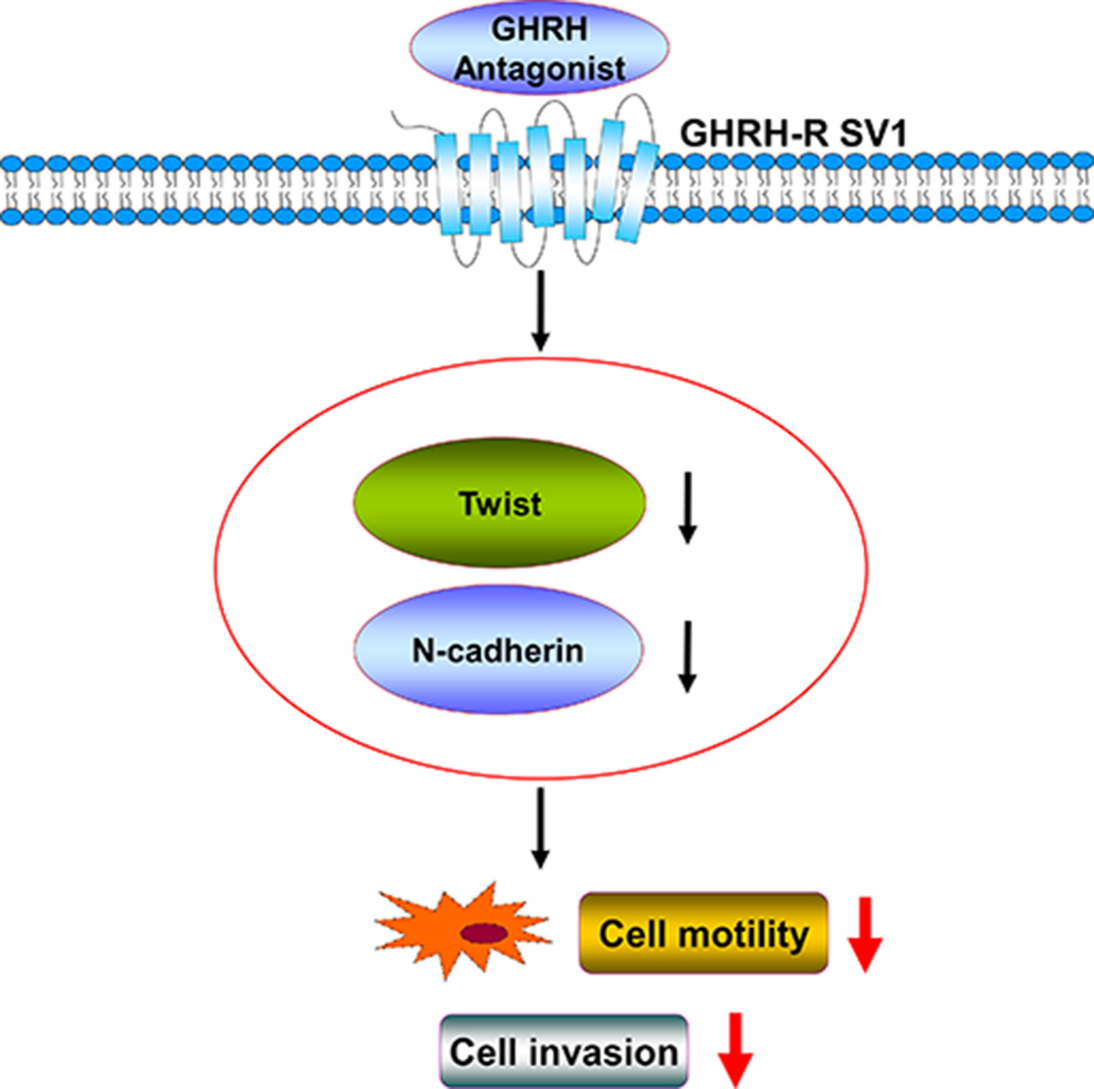
The proposed signaling pathways involved in GHRH antagonist–inhibited invasion and migration of human endometrial cancer cells by down-regulating Twist and N-cadherin expression GHRH antagonist suppresses the invasion and migration of endometrial cancer cells through binding of GHRH receptors and suppression of the Twist and N-cadherin pathways.

Since both Twist and N-cadherin were expressed in Ishikawa and ECC-1 cells and their expressions were required for maintaining the cell migration and invasion, we therefore examined whether GHRH antagonist inhibited cell migration and invasion by regulating Twist and N-cadherin expression. RT-PCR and Western blotting analyses showed that treatment with GHRH antagonist, MIA-602, significantly down-regulated mRNA and protein levels of Twist and N-cadherin in both Ishikawa and ECC-1 cells (Figure 5A and 5B). Interestingly, treatment with MIA-602 down-regulated Twist and N-cadherin protein levels in both cell lines and the inhibitory effects of MIA-602 on Twist and N-cadherin expression were abolished by the siRNA-mediated knockdown of GHRH-R (Figure 5C). In addition, MIA-602 treatment inhibited Ishikawa and ECC-1 cell migration and invasion. Moreover, knockdown of GHRH-R abolished the MIA-602-induced decreases of cell migration and invasion in both Ishikawa and ECC-1 cells (Figure 5D and 5E). These results indicated GHRH antagonist inhibited human endometrial cancer cell migration and invasion by down-regulating the expression of Twist and N-cadherin. Taken together, our data demonstrate that GHRH antagonist–inhibited invasion and migration of human endometrial cancer cells by down-regulating Twist and N-cadherin expression. GHRH antagonist suppresses the invasion and migration of endometrial cancer cells through binding of GHRH receptors and suppression of the Twist and N-cadherin pathways (Figure 6).

## DISCUSSION

Antagonists of GHRH have been shown to inhibit the cell growth and behavior of multiple types of cancer cells [4, 17–20]. Further evidence demonstrates that the antitumor effect of GHRH antagonists is mediated by the suppression of tumor growth through autocrine/paracrine faction rather than regulating the pituitary-GH-hepatic IGF-I axis [21]. Our previous studies have demonstrated the direct antitumor effects of GHRH analogues in human endometrial cancer cells [4, 6]. Therefore, GHRH antagonists have potent effects on endometrial tumors, suggesting that GHRH antagonists may be used as possible therapeutics for human endometrial cancers. It has been shown that the expressions of GHRH and GHRH-R SV1 and pituitary GHRH-R can be detected in many different types of human cancer. In addition, both GHRH-R SV1 and pituitary GHRH-R are involved in GHRH antagonists-induced antitumor effects [3, 22, 23]. In the present study, our results showed that both mRNA and protein levels of GHRH and GHRH-R SV1 were expressed in two human endometrial cancer cell lines, Ishikawa and ECC-1. Importantly, our immunohistochemistry analyses also demonstrated that GHRH-R and GHRH were expressed in the human endometrial cancer tumor samples. These results reveal important paracrine and autocrine roles for GHRH in human endometrial cancer. In the early 1990s, the clinical use of GHRH antagonists has been advocated [24, 25]. To date, several GHRH antagonists, including MIA-602, that with histidine and ornithine replacements have been shown to have potent antitumor effects [4, 15, 23, 26, 27]. However, the mechanisms of the suppression of cell migration and invasion of GHRH antagonists, such as MIA-602, in endometrial cancer cells were unresolved until the present study was performed. In the present study, we demonstrated for the first time that a GHRH antagonist, MIA-602, inhibited the cell migration and invasion of human endometrial cancer by down-regulating Twist and N-cadherin expression. The inhibitory effects of GHRH on human endometrial cancer cell migration and invasion were mediated by GHRH-R. These results provide insights into the prospect of developing targeted therapy for human endometrial cancer.

Our previous study has demonstrated that GHRH antagonist induces cell apoptosis in human endometrial cancer cells [6]. In current present study, we further showed that the treatment of human cancer cells with a GHRH antagonist inhibited the cell migration and invasion. In addition, the inhibitory effects of GHRH on human endometrial cancer cell migration and invasion were mediated by GHRH-R. These findings provide confirmation that GHRH antagonist inhibited cell motility in endometrial cancer. Our findings also agreed with the results of previous studies suggesting that GHRH antagonist may inhibit cell motility and growth in gynecologic cancer cell lines [6, 19]. Therefore, GHRH antagonist can exhibit its antitumor effects by inducing cell apoptosis and inhibiting cell migration and invasion.

N-cadherin has been showed critical in cancer metastasis, and the up-regulation of N-cadherin is involved with poor outcome in cancer [8–10]. Twist has been shown to regulate human N-cadherin expression [28]. Therefore, it is proposed that Twist is involved in the mechanisms of cell migration and invasion through the regulation of N-cadherin. Our studies demonstrate that Twist and N-cadherin are highly expressed in Ishikawa and ECC-1 endometrial cancer cells. Pre-treatment with Twist/ N-cadherin siRNA abolished the protein expression of Twist/ N-cadherin, and cell migration and invasion were decreased, suggesting that the Twist/ N-cadherin signaling pathway may activate cell migration and invasion in endometrial cancer. Therefore, Twist and N-cadherin are each functionally important for the cell motility of human endometrial cancer.

In the present study, we demonstrated that a GHRH antagonist inhibited cell migration and invasion of endometrial cancer through the decreased the expression and Twist and N-cadherin. To integrate with the previous consequences from other subprojects, cell migration and invasion in endometrial cancer are mediated by the expression of the Twist and N-cadherin, and suppressed by a GHRH antagonist through the down-regulation of Twist and N-cadherin. Our findings in the present study provide a novel concept. Our data demonstrate that targeting Twist and N-cadherin with a GHRH antagonist blocked Twist and N-cadherin-induced cell migration and invasion, specifying that the actions of a GHRH antagonist in endometrial cancer cells are highly associated with Twist and N-cadherin expression.

In conclusion, our results reveal that the potential role of a GHRH antagonist in inhibiting human endometrial cancer cell migration and invasion is through the binding of GHRH-R, and the subsequent down-regulation the expression of the metastasis-related proteins Twist and N-cadherin. These results provide a mechanistic rationale for the observed GHRH-R expression in human endometrial cancer. Our findings provide new insights into the mechanism of GHRH antagonist-induced inhibition of cell migration and invasion in endometrial cancer and suggest the feasibility of developing GHRH antagonists as potential therapeutics for the clinical treatment of human endometrial cancer.

## MATERIALS AND METHODS

### Cell lines and cell culture

The human endometrial cancer cell lines Ishikawa and ECC-1 were utilized in this study. The human endometrial cancer cell line Ishikawa is a well-differentiated endometrial adenocarcinoma cell line [29]. The ECC-1 cell line, derived from a well-differentiated adenocarcinoma of the endometrium [30], was obtained from the American Type Culture Collection (USA). The cells were cultured in Dulbecco's minimum essential medium (DMEM) with 10% fetal bovine serum (FBS; Hyclone Laboratories Inc., Logan, UT), 100 U/ml penicillin, and 100 μg/ml streptomycin and incubated at 37°C in a humidified incubator with 5% CO_2_. The cells were grown to 80% confluence and transferred to serum-free medium for 24 h prior to treatment with the GHRH antagonist.

### Reagents

The GHRH antagonist, MIA-602, was synthesized in the US laboratory of A.V.S. by solid-phase methodology using Boc-chemistry as previously described [4, 31]. MIA-602 has the sequence: [(PhAc-Ada)^0^-Tyr^1^, D-Arg^2^, Fpa5^6^, Ala^8^, Har^9^, Tyr(Me)^10^, His^11^, Orn^12^, Abu^15^, His^20^, Orn^21^, Nle^27^, D-Arg^28^, and Har^29^] hGHRH(1–29)NH_2_, where Ada is 12-aminododecanoic acid, Fpa5 is pentafluoro-phenylalanine, Orn is ornithine, Har is homoarginine, Nle is norleucine, PhAc is phenylacetyl, and Tyr(Me) is O-methyl-Tyr. For use, stock solutions of MIA-602 were dissolved in dimethyl sulfoxide (DMSO) at a concentration of 1 μM. The concentration of DMSO after dilution in the incubation medium was low (< 0.1%) to avoid any DMSO influence on cell culture.

### Western blotting

The cells were lysed in buffer containing 20 mM Tris, pH 7.4; 2 mM EGTA; 2 mM Na_2_VO_3_; 2 mM Na_4_P_2_O_7_; 2% Triton X-100; 2% SDS; 1 μM aprotinin; 1 μM leupeptin and 1 mM PMSF. The protein concentration was determined with a protein assay kit using BSA standards according to the manufacturer's instructions (Bio-Rad Laboratories, Hercules, CA). Equal amounts of cell lysate were separated by SDS polyacrylamide gel electrophoresis (PAGE) and transferred to a nitrocellulose membrane (Hybond-C, Amersham Pharmacia Biotech Inc., Oakville, ON). Following blocking with Tris-buffered saline (TBS) containing 5% non-fat dry milk for 1 h, the membranes were incubated overnight at 4°C with anti-GHRH-R (Abcam, Cambridge, MA), anti-Twist (Thermal), or anti-N-cadherin (Millipore) antibody, followed by incubation with a HRP-conjugated secondary antibody. The immunoreactive bands were detected with an enhanced chemiluminescence (ECL) kit. The membrane was then stripped with stripping buffer (62.5 mM Tris, 10 mM DTT, and 2% SDS, pH 6.7) at 50°C for 30 min and re-probed with an anti-GAPDH antibody (Santa Cruz) as a loading control.

### Immunohistochemistry (IHC)

To determine the expression of the GHRH-R and GHRH proteins in human endometrial cancer, IHC was performed on sections of human endometrial cancer tissue using previously reported procedures [32]. The involvement of human subjects in this study was approved by the Institutional Review Board of Chang Gung Memorial Hospital (CGMH-IRB numbers 101-2187B and 100–3879C). Four-micrometer-thick formalin-fixed, paraffin-embedded (FFPE) tissue sections were deparaffinized in xylene and rehydrated with a graded series of ethanol solutions. The sections were then stained with anti-human GHRH-R and GHRH polyclonal antibodies (Abcam, 1:100) using an automated IHC stainer with the Ventana Basic DAB Detection kit (Tucson, AZ) according to the manufacturer's protocol. Counterstaining was performed with hematoxylin. Sections were stained without the GHRH-R and GHRH antibodies as a negative control in the fourth of four columns depicting the human endometrial cancer tissue sections.

### Small interfering RNA transfection

siGENOME ON-TARGETplus SMARTpool human GHRH-R siRNA, Twist siRNA, N-cadherin siRNA and siCONTROL NON-TARGETINGpool siRNA were purchased from Dharmacon. The cells were transfected with siRNA (20 nM) using Lipofectamine RNAiMAX. After a 24 h transfection, the medium was removed and changed to fresh serum-free medium.

### Invasion and migration assays

Migration and invasion assays were performed in Boyden chambers with minor modifications. Cell culture inserts (24 -well, 8 μm pore size; BD Biosciences, Mississauga, ON) were seeded with 1 × 10^5^ cells in 250 μl of medium with 0.1% FBS. Un-coated inserts were used for the migration assays, whereas inserts pre-coated with growth factor-reduced Matrigel (40 μl, 1 mg/ml; BD Biosciences) were used for invasion assays. Medium with 10% FBS (750 μl) was added to the lower chamber and served as a chemotactic agent. After 24 h (migration) or 48 h (invasion) incubation, non-migrating/invading cells were wiped from the upper side of the membrane and cells on the lower side were fixed in cold methanol (−20°C) and air dried. In these assays, the cells that had not penetrated the filter were removed by wiping, and the cells that had invaded the lower surface of the filter were fixed with ice-cold methanol and stained with 0.5% crystal violet.

### RT-nested PCR for detection of GHRH and SVs of GHRH-R

Total RNA was extracted from cultured Ishikawa, ECC-1, T47D and HeLa cells using TRIzol reagent (Invitrogen). The total RNA concentration was determined from spectrophotometric analysis at A260/280. Single-stranded complementary.

DNA (cDNA) was synthesized from 2 μg of total RNA by reverse transcription (RT) (Amersham Biosciences, Baie d’Urfe’, Quebec, Canada). The synthesized cDNA was used as a template for PCR amplification. To investigate the presence of GHRH-R SVs and to improve the specificity and sensitivity of amplification, nested PCR was performed as described previously [6, 33].

### Statistical analysis

The results are presented as the mean ± SEM of at least three independent experiments. GraphPad Prism software was used for statistical analysis. Multiple comparisons were analyzed by one-way ANOVA followed by Tukey's multiple comparison tests. A significant difference was defined as *p* < 0.05.
